# Evaluation of local oxygen flux produced by photoelectrochemical hydroxide oxidation by scanning electrochemical microscopy

**DOI:** 10.1038/s41598-023-32210-6

**Published:** 2023-03-28

**Authors:** Bhavana Gupta, Ariba Aziz, Shashank Sundriyal, Vishal Shrivastav, Ambrose A. Melvin, Marcin Holdynski, Wojciech Nogala

**Affiliations:** Institute of Physical Chemistry, Polish Academy of Sciences, Kasprzaka 44/52, 01-224 Warsaw, Poland

**Keywords:** Chemistry, Energy science and technology, Materials science

## Abstract

Several in-situ electrochemical approaches have been developed for performing a localized photoelectrochemical investigation of the photoanode. One of the techniques is scanning electrochemical microscopy (SECM), which probes local heterogeneous reaction kinetics and fluxes of generated species. In traditional SECM analysis of photocatalysts, evaluation of the influence of radiation on the rate of studied reaction requires an additional dark background experiment. Here, using SECM and an inverted optical microscope, we demonstrate the determination of O_2_ flux caused by light-driven photoelectrocatalytic water splitting. Photocatalytic signal and dark background are recorded in a single SECM image. We used an indium tin oxide electrode modified with hematite (α-Fe_2_O_3_) by electrodeposition as a model sample. The light-driven flux of oxygen is calculated by analysis of SECM image recorded in substrate generation/tip collection mode. In photoelectrochemistry, the qualitative and quantitative knowledge of oxygen evolution will open new doors for understanding the local effects of dopants and hole scavengers in a straightforward and conventional manner.

## Introduction

A variety of catalysts have been tried to lower the electrical energy needed for water splitting in the case of renewable energy generation using water electrolysis in the presence of light^1,2^. When performing a hydrogen evolution reaction (HER), the overall process (water splitting) rate is often limited by the rate of the process occurring on the second electrode. Therefore, photoanode efficiency is essential. Since the oxidation of water is thermodynamically more challenging than the reduction of water into hydrogen, research into photoanodes is more encouraged^3^. By demonstrating reduced overpotential and/or higher photocatalytic current, many types of materials in their unaltered or modified forms have been assessed for effective water oxidation^4–6^. Efficient engineering of water splitting photoanodes, besides traditional photoelectrochemical measurements, requires fast in-situ surface characterization.

One of the in-situ electrochemical techniques, scanning electrochemical microscopy (SECM), has been utilized to analyze photoanode surfaces locally^7–14^. Different approaches have been used to develop the SECM technology, particularly in the lighting method, which allows for simple and straightforward surface analysis with a high spatial and temporal resolution, starting from illuminating a large area of analyzed photocatalysts to local illumination of SECM-analyzed area by laser or using SECM tip as optical fiber^14^. Numerous flaws, including partially shadowing the photoactive surface with the ultramicroelectrode and easy supersaturation of the electrolyte with the product gas, are revealed by larger-scale lighting, especially from the top side. Different updates to the SECM setup were made, particularly with microelectrodes for local lighting. Ring-shaped microelectrode design with coaxial internal optical fiber for local illumination limits the resolution of electrochemical imaging^10,14,15^. Additionally, this method of microelectrode modification takes time and requires a complex fabrication process. Another method of local illumination of the SECM-analyzed sample to avoid shadowing by the SECM tip is the delivery of light through the insulating glass sheath of the microelectrode^16^. This approach allows recording the sample photocurrent and the tip current corresponding to the faradaic collection of the product (e.g., O_2_) of the photocatalytic reaction generated at the sample, both as a function of the lateral probe position. However, retrieving quantitative information on the local flux of generated products is challenging. Oxygen evolution delivers quantitative information about the photoelectrochemical process in the form of the flux of generated product. Thus, it is imperative to measure evolved oxygen individually and quantitatively to comprehend the true efficiency of the photoanode. With the use of an SECM tip electrode, a more straightforward surface analysis method of the photoanode that offers O_2_ photogeneration qualitatively and quantitatively is required.

Here, we demonstrate a simple method for localized photoanode lighting within the SECM scanning area that provides quantitative data on locally photogenerated oxygen flux. It relies on using an inverted optical microscope coupled with a light source. We demonstrate SECM analysis of a semi-transparent α-Fe_2_O_3_ covered ITO substrate illuminated locally from the back side. α-Fe_2_O_3_ is known for its photocatalytic activity towards O_2_ generation in alkaline media without producing H_2_O_2_^4^. By adjusting the objective lens magnification and/or the aperture size, this technique can provide illumination throughout a wide to a narrow range and allows for fast and easy positioning of the SECM tip over the area of interest at the sample.

## Experimental

### Preparation of α-Fe_2_O_3_ by galvanostatic electrochemical deposition

A solution of FeCl_3_ (Chempur 25 mM) and FeCl_2_ (Chempur 15 mM) in water was produced for the deposition of α-Fe_2_O_3_ on the surface of ITO coated glass (Visiontek Systems Ltd.). Ag/AgCl/KCl(3 M) served as the reference electrode, and the steel plate served as the counter electrode. For cathodic deposition on the (1.5 × 1) cm^2^ ITO working electrode, a continuous current of − 1.5 mA was applied to the working electrode for 200 s^17^. After deposition, α-Fe_2_O_3_ covered ITO glass was rinsed with demineralized water (~ 18 MΩ·cm), dried under ambient air and annealed at 500 °C for 1 h.

### Characterization of α-Fe_2_O_3_ film

Synthesized films were characterized using SEM (FEI Nova NanoSEM 450 outfitted with GENESIS software and an EDX detector). The XRD pattern was captured using PANalytical Empyrean diffractometer fitted with a X'Celerator detector using Ni-filtered Cu Kα radiation (λ_1_ = 1.54056 Å and λ_2_ = 1.54439 Å). Data were collected on a flat plate θ/θ geometry on a spinning sample holder. X-ray photoelectron spectroscopy (XPS) observations were carried out using a Microlab 350 (Thermo Electron) spectrometer with a lateral resolution of 0.2 mm^2^ and Al-K non-monochromated radiation (1486.6 eV; 300 W) as the excitation source. The analysis was conducted at a pressure of 5.0 × 10^−9^ mbar. High resolution and survey spectra were captured utilizing pass energies of 40 and 100 eV, respectively. Measurements of the photoelectrochemical process were made in a cell with a photoanode fixed at its base. Ag/AgCl/KCl(3 M) and Pt wire were used as a reference and counter electrode on top of the working electrode. The electrolyte was 0.5 M NaOH (Chempur). White light irradiation of intensity of LED (pE-300white) light outfitted with shamrock filters was used.

### Scanning electrochemical microscopy for photoelectrochemical measurement

**Figure 1:**
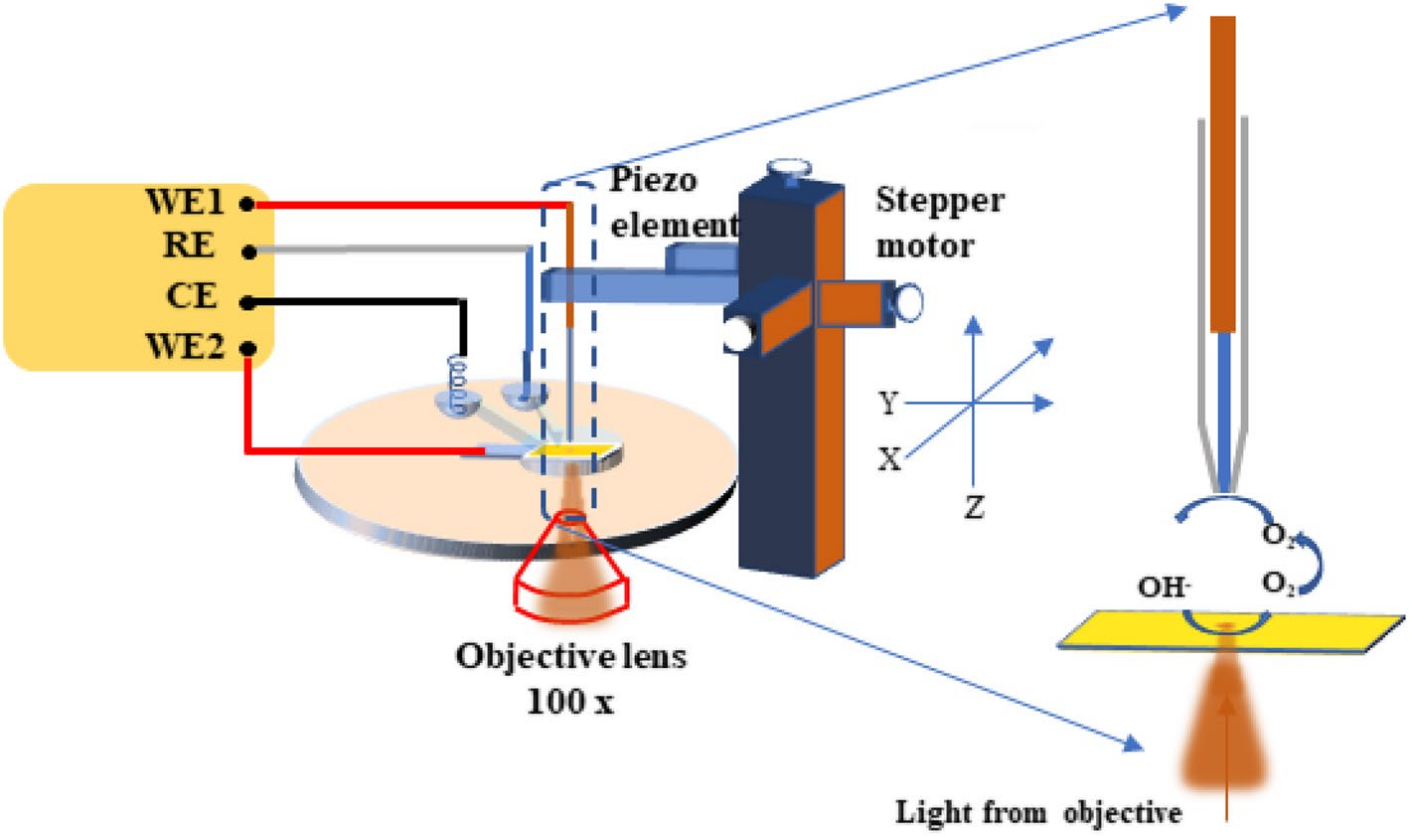
Schematic illustration of SECM setup and zoomed part of α-Fe_2_O_3_ substrate and microelectrode showing photoelectrochemical and oxygen reduction reaction, respectively.

A home-build SECM setup composed of closed-loop piezo actuators (P625.2CD and P622.1CD, PI HERA with LVPZT Amplifier E-509 C3A, PhysikInstrumente), two VA-10X patch-clamp amplifiers (npi Electronic Instruments), operated under SECMx software^18^ through PCIDAS1602/16 analog–digital (AD) and PCI-DDA04 DA digital-analog (DA) cards (Measurement Computing) was installed on top of an inverted optical microscope Nikon MA200 with 150 W Tungsten lamp using a homemade adapter. Two patch-clamp amplifiers with three electrode preamplifiers were connected in a bipotentiostatic mode for independent polarization of the sample and the SECM tip and current measurement. Platinum was employed as a counter electrode, while Ag wire served as a quasi-reference electrode in SECM measurements. The photoanode substrate was mounted at the bottom of a Teflon cell. Its surface area contacting with the electrolyte was confined to a circle with a diameter of one millimeter to prevent current overflow (max. 2 µA for used equipment). 0.5 M NaOH was employed as an electrolyte. The sample was illuminated locally through a long working distance (4.5 mm) 100 × objective (NA = 0.8), as shown in Fig. 1. Two sizes of Pt disk microelectrodes (diameter 25 µm and 4 µm) were used as SECM tips. The distance between the Pt tip microelectrode and the semitransparent α-Fe_2_O_3_ substrate was adjusted by approaching (touching) and retracting method with the help of the inverted optical microscope^19^. Narrow depth of field (< 1 µm) for 100 X objective allowed for precise focusing on the sample or on the tip. The micrometric screw of the microscope has a scale with 1 µm precision. The tip-to-sample distance can be measured by subtracting the micrometric screw positions when focused on the sample and on the tip. The tip insulating glass sheath was positioned to be in contact with the α-Fe_2_O_3_ substrate and then it was retracted 10 µm using the piezo motor. The distance of 10 µm was verified by optical reading. Accordingly, it is projected that the light intensity reaching the substrate will be 25, 35, and 45 mW cm^−2^ for objective lenses with magnifications of 100 ×, 50 ×, and 20 ×, respectively. SECM data analysis was carried out using the MIRA software^20^.

## Results and discussion

**Figure 2:**
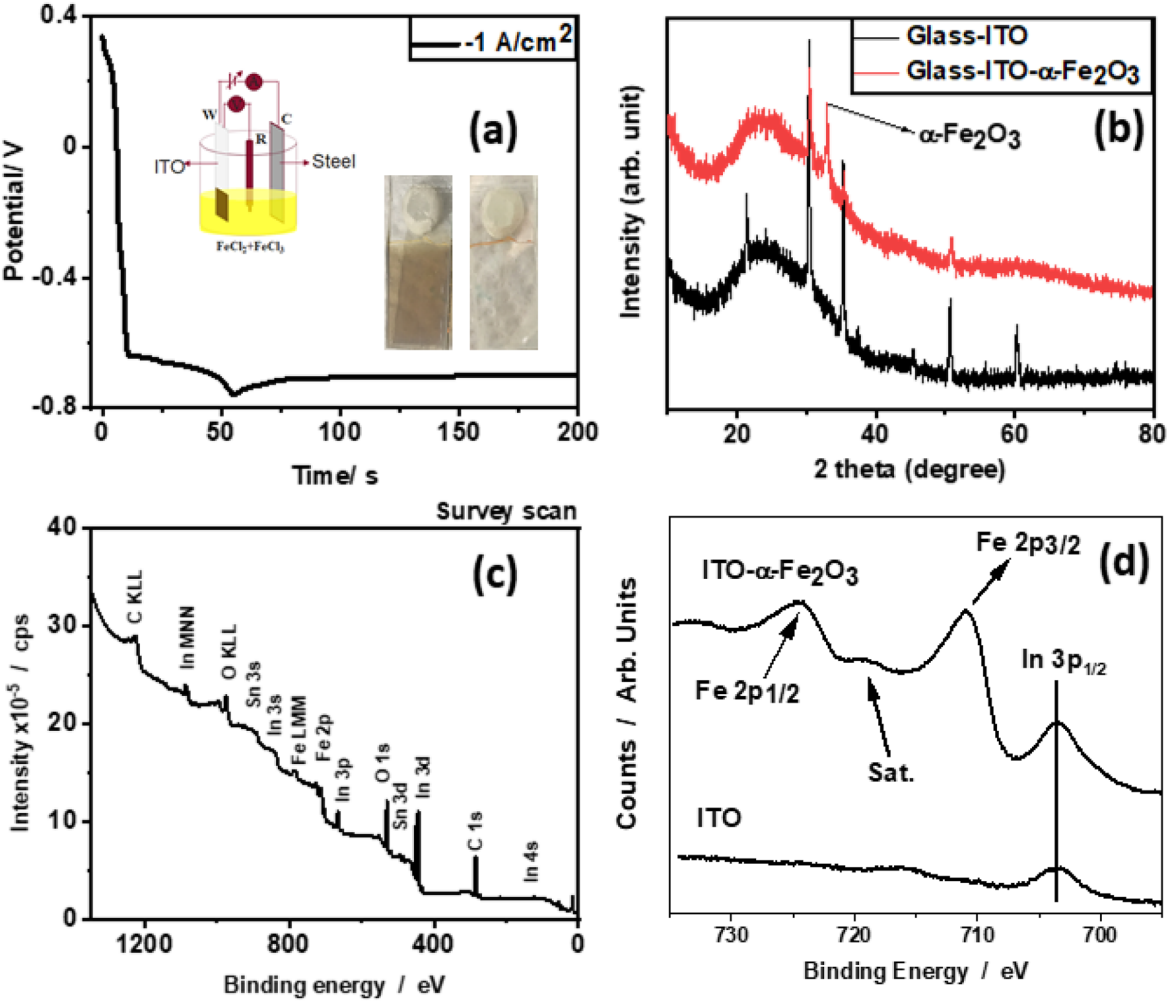
(**a**) Chronopotentiometry curve of α-Fe_2_O_3_ deposition at − 1 mA cm^−2^ current density. (Inset-optical images of the resultant film before annealing (left) and after annealing (right)), (**b**) XRD patterns of ITO-glass and α-Fe_2_O_3_ on ITO-glass, (**c**) XPS survey spectrum of α-Fe_2_O_3_ on ITO-Glass and (**d**) XPS spectra of In 3p and Fe 2p.

Electrolytic preparation of α-Fe_2_O_3_ takes place by cathodic deposition Fig. 2a^17^. The process consists of the precipitation of Fe^3+^ with HO^−^ to form Fe(OH)_3_, and then the formation of Fe_2_O_3_ from the evaporation of H_2_O during the annealing process:

1 $$2{\text{H}}_{2} {\text{O}} + 2{\text{e}}^{ - } \to {\text{H}}_{2} + 2{\text{OH}}^{ - }$$

2 $${\text{Fe}}^{3 + } + 3{\text{OH}}^{ - } \to {\text{Fe}}\left( {{\text{OH}}} \right)_{3}$$

3 $$2{\text{Fe}}\left( {{\text{OH}}} \right)_{3} \to {\text{ Fe}}_{2} {\text{O}}_{3} + 3{\text{H}}_{2} {\text{O}}{.}$$

On ITO, water reduction and the change of ferric ions into ferrous ions happen concurrently. Water reduction alters the local pH at the electrode, causing hydroxide to precipitate. There is a decline in current with time and finally, saturates supports the complete participation of the surface in the electrochemical deposition. From an electrochemistry point of view, it is called the charging process. The first regime (0 to ~ 10 s) in the chronopotentiometry in Fig. 1a is due to oxygen depletion near the electrode. Later, > ca. 10 s, water reduction starts (Eq. 1), which requires growing overpotential due to local alkalization near the electrode. After ca. 55 s, iron hydroxide is formed and the potential stabilizes. The production of hematite is also aided by oxygen reduction at the cathode, causing a local increase in pH. Galvanostatic cathodic electrodeposition causes the creation of a thin, brown color coating on the surface of transparent ITO (Fig. 2a-Inset-left image). After annealing, a brown color film turns into a translucent yellowish-orange film, most likely as a result of moisture evaporation and the conversion of hydroxide to oxide (Fig. 2a-Inset-right image). X-ray diffraction was used to characterize the finished product after annealing. Glass and ITO were employed as a reference for the substrate in order to verify the product formation. A prominent peak at 33.15°^21^ and the suppression of the ITO peak at 21.25°, 30.39°, 35.35°, 50.76°, and 60.25° are evidence of the thin film deposition of Fe_2_O_3_ (Fig. 1b). Due to the lack of crystallinity, the quality of the broad peak at 24.5°, which is related to glass, remains unchanged. Various elements seen in the general survey XPS spectrum, including Fe and O, are derived from α-Fe_2_O_3_, while In, Sn and O are components of ITO (Fig. 2c). In addition, XPS could also detect C as a surface impurity. Two major peaks, corresponding to Fe 2p_3/2_ and Fe 2p_1/2_, are seen as a distinctive property of Fe at binding energies of 710.93 and 724.6 eV, respectively (Fig. 2d).

**Figure 3:**
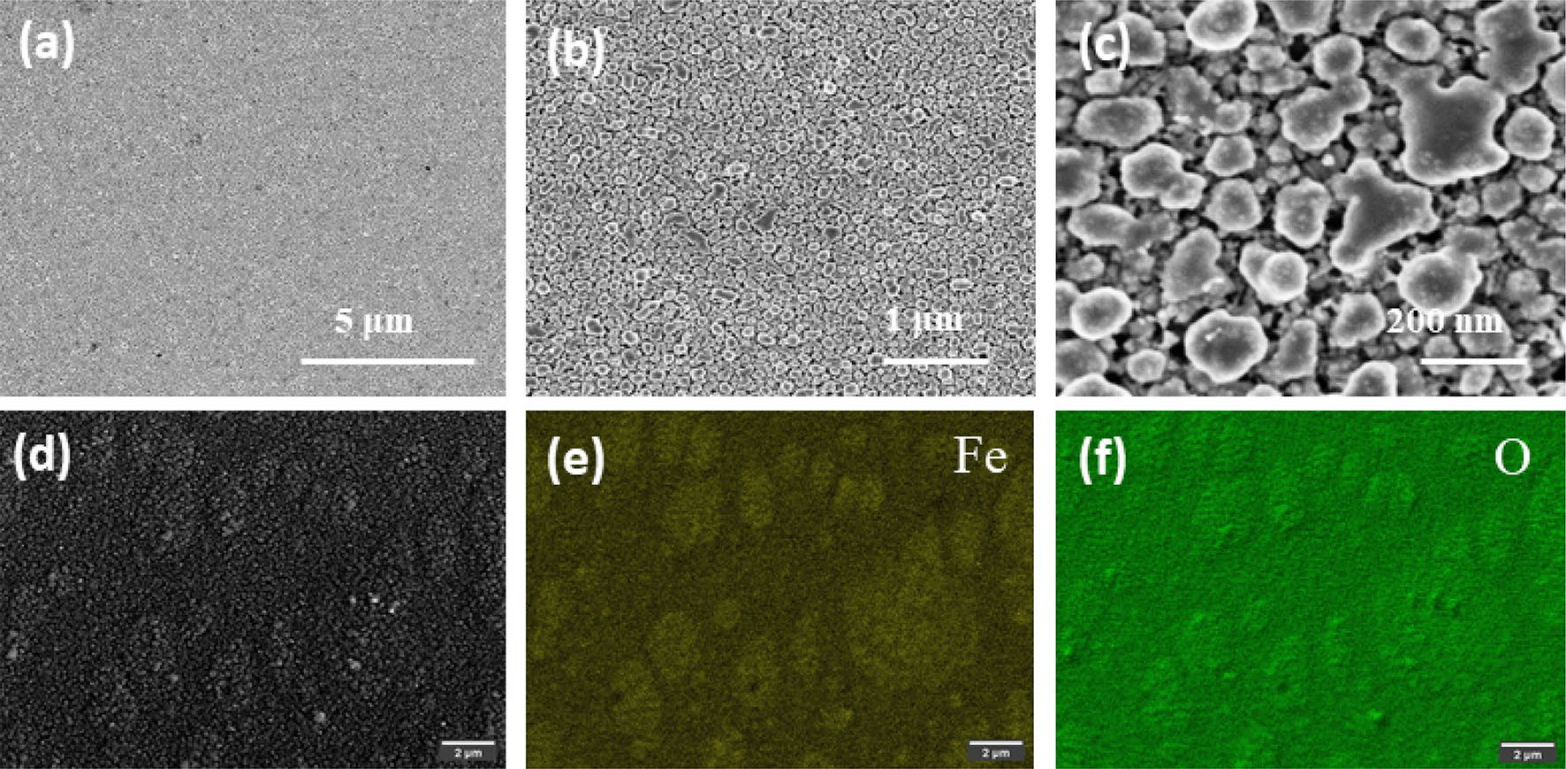
(**a**–**c**) SEM images of α-Fe_2_O_3_ with different magnifications, (**d**–**f**) SEM image (**d**) and EDS maps of Fe (**e**) and O (**f**).

The surface of α-Fe_2_O_3_ was studied using scanning electron microscopy. From low to high magnification of SEM pictures, the film's surface is very uniform with granular morphology, with each granule having a nanoscale size (Fig. 3a–c). Fe and O elements are clearly present in the surface’s EDS mapping (Fig. 3d–f).

**Figure 4:**
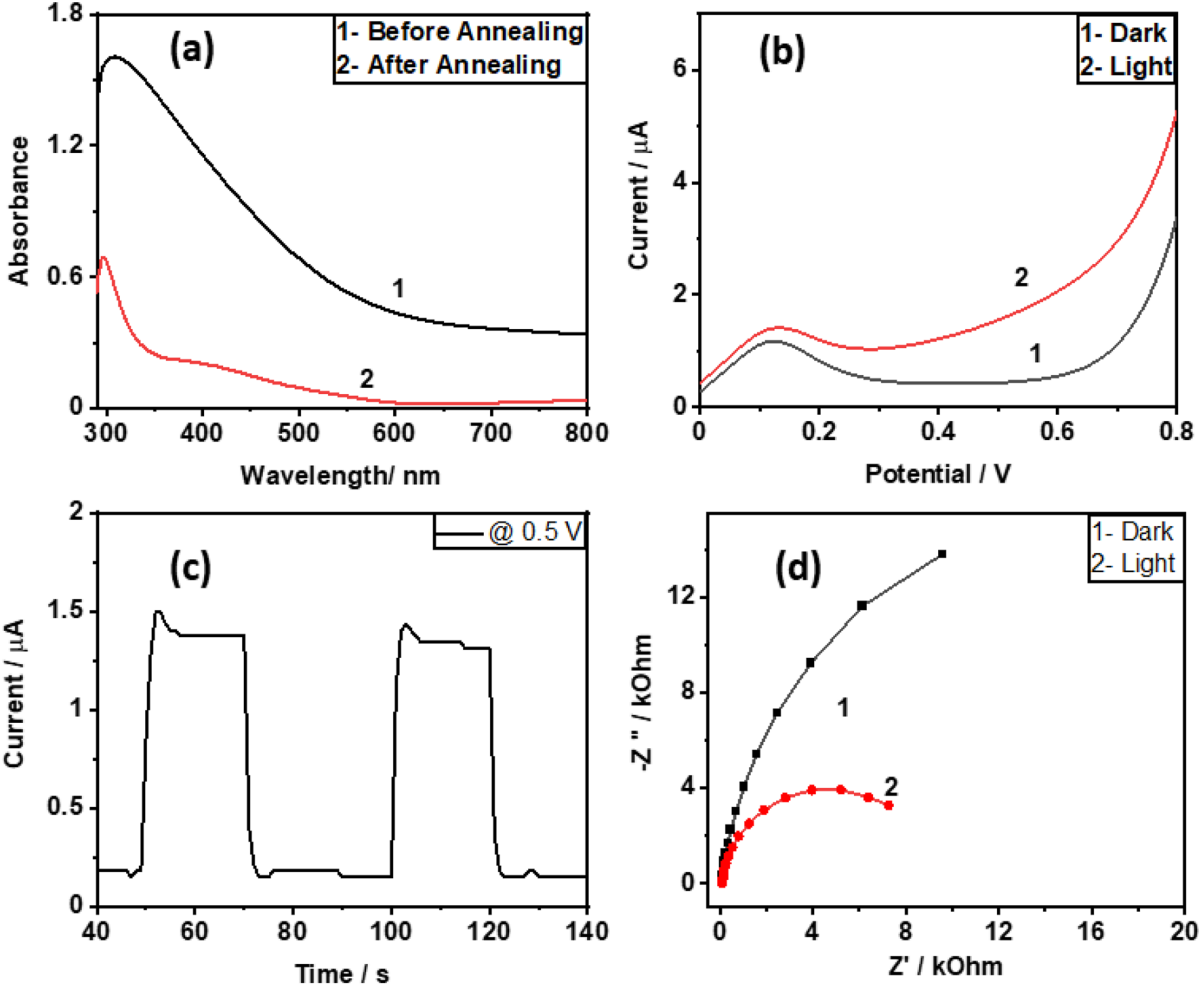
(**a**) UV–Vis spectra of α-Fe_2_O_3_ before (1) and after (2) annealing, (**b**) linear sweep voltammetry curve under dark (1) and light (2) conditions c) Chronoamperometric curve of α-Fe_2_O_3_ under the light on–off conditions at 0.5 V potential and d) Nyquist plot under the dark (1) and light (2) condition at in 0.5 M NaOH 0.1 Hz to 10 kHz frequency range and 5 mV AC RMS amplitude. The area of the exposed electrode is 1.5 cm^2^.

The resulting layer from electrochemical deposition was examined by UV–vis spectroscopy to check its light absorbance. After annealing, it was noticed that the film became less opaque in the entire analyzed spectrum, as depicted in Fig. 4a. Moreover, the spectrum reveals a clear absorption peak (350–550 nm), which supports distinct and desired band gap generation. The film can generally absorb a wide range of wavelengths with a distinct band gap, which is crucial for converting light energy into fuel and/or power. Photoelectrochemistry of the resulting film was performed in 0.5 M NaOH under UV–Vis irradiation. According to Fig. 4b, linear sweep voltammetry (LSV) as a photoelectrochemical test reveals considerable photocurrent for potentials higher than ca. 0.3 V. In the presence of light, the onset potential for water splitting is switched^22^. The water splitting onset potential is 0.5 V in complete darkness and shifts to 0.35 V in light. Chronoamperometry demonstrates a six-fold rise in current in the presence of light (Fig. 4c) that is coherent with the LSV result, confirming photocatalytic reaction at 0.5 V.

Impedance spectroscopy analysis reveals the greater charge transfer feasibility of α-Fe_2_O_3_ in the presence of light compared to the dark condition, validating the results of LSV and chronoamperometry (Fig. 4d). Photoelectrochemistry results support the activity of photoanode for light-induced water oxidation. This property and transparency of α-Fe_2_O_3_ encouraged us to analyze localized photoactivity with SECM.

**Figure 5:**
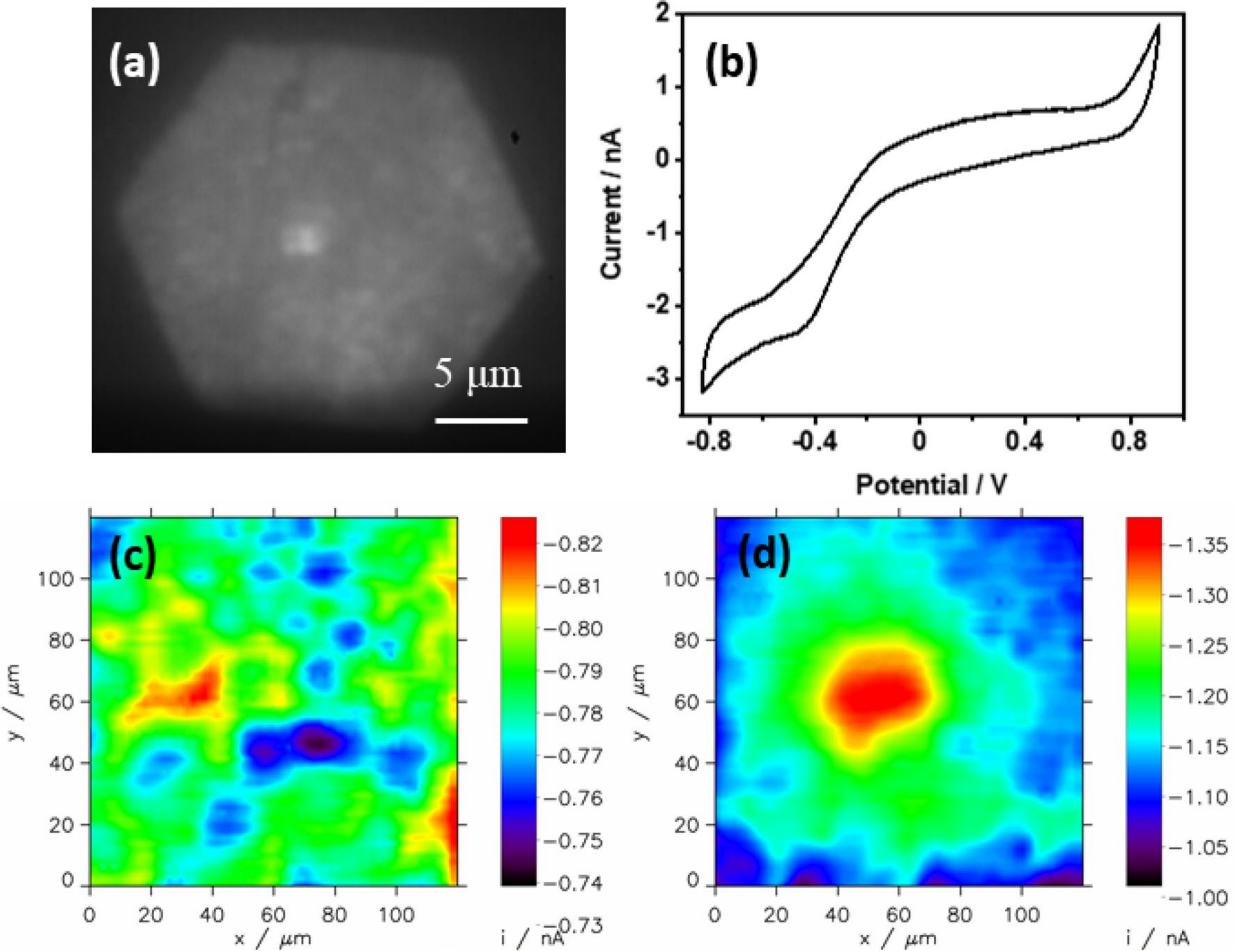
(**a**) Optical image of α-Fe_2_O_3_ substrate illuminated with 100 × objective with closed hexagonal aperture diaphragm. The brighter blurred spot is Pt microelectrode of 2 μm radius positioned 10 µm above the sample. (**b**) Cyclic voltammogram of the microtip in 0.5 M NaOH at a scan rate of 25 mV/s. (**c**) SECM scan of illuminated floating (without applying any bias) sample. (**d**) SECM scan with illumination in the biased condition to the photoanode. Electrolyte: 0.5 M NaOH, sample potential: 0.7 V, tip potential − 0.6 V. Scanning rate: 10 µm/s.

The initial surface of the substrate, which has an exposure to light of approximately 35 μm, was focused in order to analyze the surface of the α-Fe_2_O_3_ substrate using SECM (Fig. 5a). Microelectrodes with a 2 μm radius are getting close enough to each other to be roughly 10 μm apart.

The activity of the microelectrode was checked by cyclic voltammetry (Fig. 5b). It was found that its potential appropriate for diffusion limiting oxygen reduction without water reduction is in the range of − 0.4 V to − 0.6 V. For potentials more negative than − 0.6 V hydrogen evolution starts, which should be avoided in further SECM experiments. The surface was initially scanned while the substrate (photoanode) was not polarized (disconnected from the current amplifier). The inactive sample was hindering the diffusion of oxygen to the SECM tip. According to Fig. 5c, the surface is heterogeneous in terms of topography. Similar inhomogeneities are visible in SEM micrographs (Fig. 3d). The microscopic inhomogeneities are also visible with optical microscopy (Fig. 5a). The product of 4-electron reduction of oxygen at the tip is water, i.e. the solvent, whose concentration does not change locally due to the tip reduction. The little tip current variations of ca. 80 pA at the tip microelectrode are caused by little variations in the hematite layer height. No distinct signal is visible for the tip positions over the illuminated area. This shows that only illumination, without electrical bias, is not able to induce oxygen evolution. Application of bias (+ 0.7 V) to the photoanode together with light clearly shows a photoactive zone of oxygen evolution corresponding to a hexagonal shape of illuminated area (aperture diaphragm used for confining illuminated sample area to prevent flares and contrast loss caused by light reflected from the sample out of observed field in metallography). The remaining part beyond the highly illuminated hexagonal shape area is also partially illuminated by the scattered light, so the cathodic tip currents corresponding to the reduction of oxygen generated at the sample are higher than for the unbiased sample. Sluggish oxygen generation also occurs on a non-illuminated but polarized at + 0.7 V sample (Fig. 5b). Inhomogeneities are also visible for polarized photoanode (Fig. 5d).

**Figure 6:**
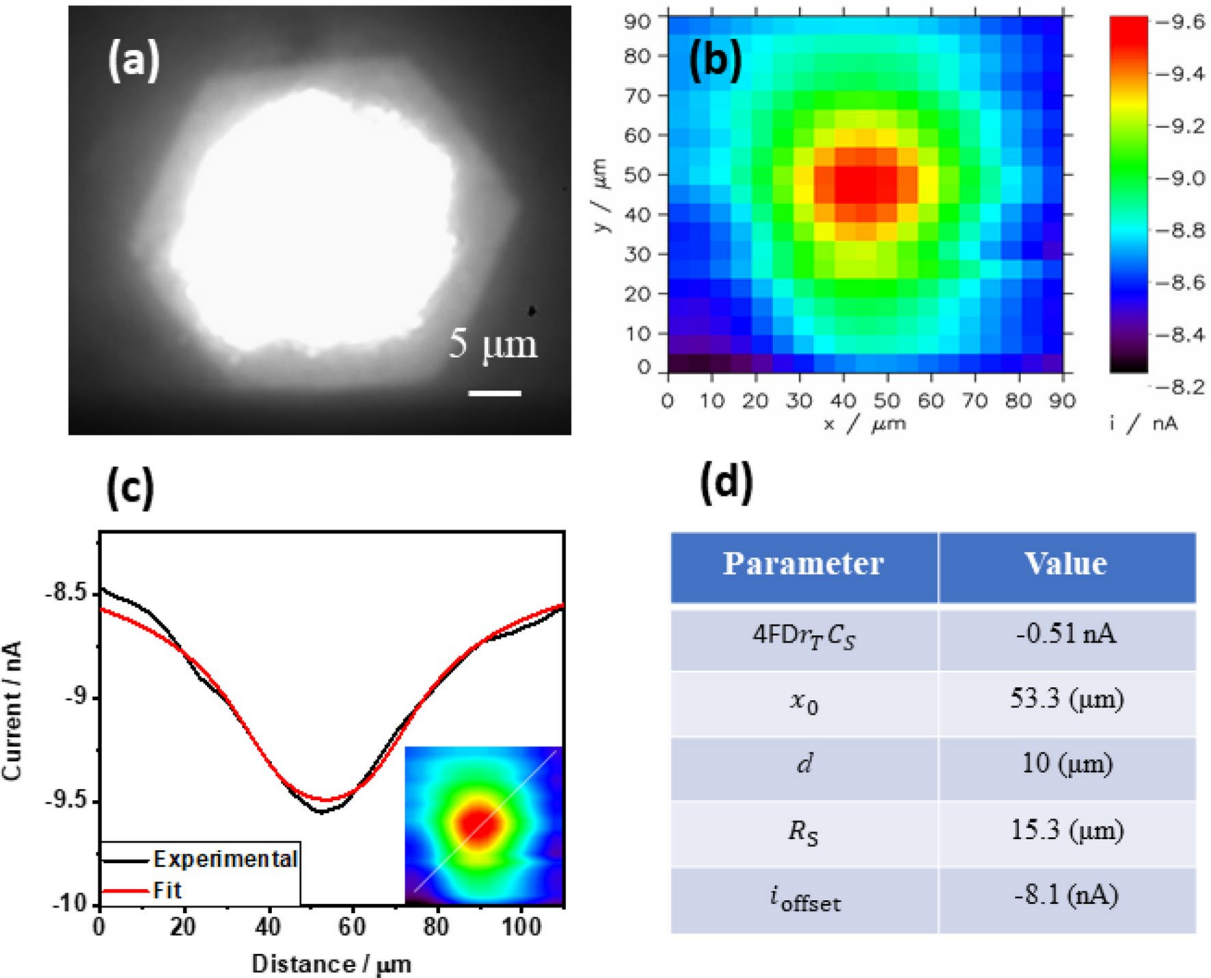
(**a**) Optical image of α-Fe_2_O_3_ substrate illuminated with 100 × objective with closed hexagonal aperture diaphragm. The brightest part corresponds to light reflected from a Pt microelectrode of 12.5 μm (radius) positioned 10 µm above the sample. (**b**) SECM image (SG/TC mode) of the illuminated part of the α-Fe_2_O_3_ sample. Electrolyte: 0.5 M NaOH, sample potential: + 0.7 V, tip potential: − 0.6 V, scanning rate: 10 µm/s. (**c**) Experimental and fitted cross-section indicated Inset interpolated image of (**b**) with white line showing cross-section extraction, (**d**) table represent fitted parameters.

While a broader area scan employing a small Pt microelectrode provides higher lateral resolution and is helpful in understanding surface heterogeneity, a bigger microelectrode provides higher sensitivity and collection efficiency, being more practical for quantitative information. In order to estimate the flux of photogenerated oxygen, the illuminated surface area was scanned using a Pt microelectrode with a 12.5 µm radius of metallic component. SECM scanning was performed 10 µm above the α-Fe_2_O_3_ substrate illuminated in the same manner as in the experiment described above. The microelectrode surface is visible when passing above the illuminated substrate during the scanning process (Fig. 6a) (supporting information Video S1 20 × accelerated). The recorded cathodic tip current is highest for the tip position above the center of the illuminated spot. It decreases as the microelectrode advances beyond the illuminated area, almost identical to the current recorded without illumination. Despite lower lateral resolution when scanning with a larger microelectrode, the averaged signal resembles a circular, micrometer-sized spot scanned with substrate generation/tip collection mode, as shown in Fig. 6b^23,24^.

A horizontal line scan over the center of the illuminated part of the sample was extracted from the interpolated image (Fig. 6c).

The extracted curve was fitted to the following equation for a horizontal line scan over a microdisk source of the generated analyte^25^ here oxygen:

4 $$i_{{\text{T}}} = 4nFDC_{{\text{S}}} r_{{\text{T}}} \xi$$

5 $$\xi = \frac{2}{\pi }actan \frac{{\sqrt 2 r_{{\text{S}}} }}{{\left( {\sqrt {\Delta x^{2} + d^{2 } - r_{{\text{S}}}^{2} \sqrt {\left( {\Delta x^{2} + d^{2} + r_{{\text{S}}}^{2} } \right)^{2} } } + 4d^{2} r_{{\text{S}}}^{2} } \right)}}$$

Fitting the data to the theoretical model with the help of MIRA software results in the surface concentration of evolved oxygen at the illuminated microspot of the sample (*C*_S_). Oxygen reduction is a 4-electron process on the microelectrode, hence *n* = 4, the diffusion coefficient of oxygen (*D*) is 1.76 × 10^−5^ cm^2^ s^−1^^26^. $r_{{\text{T}}}$ is the radius of the microelectrode, i.e., 12.5 μm. $\xi$ is the dimensionless factor describing a change in oxygen concentration along vertically (*d*—distance) and horizontally (Δ*x* = *x*-*x*_0_) from the center of the spot. Non-linear least squares fitting results in the following parameters: *nFDr*_T_*C*_S_ =  − 0.51 nA, $x_{0}$ = 53.3 µm, *r*_S_ (radius of illuminated spample area) = 15.3 µm, *i*_offset_ =  − 8.1 nA. The vertical distance between the substrate and the microelectrode was fixed at *d* = 10 µm (not fitted). The value of offset current *i*_offset_ represents the reduction of oxygen initially present in the electrolyte and also generated at the non-illuminated part of the sample polarized at + 0.7 V. Thus, the obtained parameter *C*_S_ is an excess concentration of photogenerated oxygen. This excess of surface concentration of oxygen is 60 µM. Based on the following equation, the value of oxygen flux can be calculated:

6 $$\Omega = 4Dr_{S} c_{s} = {6}.{3} \times {1}0^{{ - {15}}} \;{\text{mol}}\;{\text{s}}^{{ - {1}}}$$

Assuming the uniform flux along the illuminated region, the generation rate can be calculated from the following equation

7 $$J = \frac{\Omega }{{\pi r_{s}^{2} }} = {1}.{32}\;{\text{nmol}}\;{\text{s}}^{{ - {1}}} \;{\text{cm}}^{{ - {2}}} .$$

The oxygen flux corresponding to ~ 1.2 µA/cm^2^ at 0.7 V for a large illuminated photoanode (Fig. 3b, dark current subtracted), according to the Faraday laws of electrolysis, it ca. 3.1 pmol s^−1^ cm^−2^ (ca. 426 times lower). It is difficult to compare these fluxes because different light sources were used for sample illumination. In the case of local micrometer-size illumination with SECM, hemispherical mass transport of reactants versus linear with the larger electrode can also play a role. This method of estimating the excess concentration of photogenerated oxygen and its flux is very convenient for the assessment of light contribution to the overall process at the photoanode. Due to the small size of the illuminated area of the sample, the hemispherical diffusion of generated oxygen causes convenient mass transport conditions. This is especially important for high rates of oxygen evolution, where at larger samples, oversaturation and formation of the gas phase are problematic. This method can also be convenient for batch analysis of sets of small samples.

## Conclusions

We presented a simple technique for local probing of photoelectrochemical processes. The basic idea behind these techniques is SECM analysis of (semi)transparent samples illuminated locally using an inverted optical microscope. The obtained local excess concentration of photogenerated oxygen and its flux are related to illumination only. Background signal from oxygen present in the electrolyte and generated at non-illuminated parts of the sample is subtracted in the fitting procedure. The effectiveness of photocatalysis was precisely characterized by quantitative data on oxygen evolution. Although we presented this method on an example of a photoanode, it can also be applied for the analysis of photocathodes and interfaces active towards photoreactions (*e.g.*, photosynthesis) which are not electrodes. We suggest an SECM setup with nanoelectrodes and focused laser radiation to localize the photoactive parts of nanostructured materials.

## Supplementary Information

Supplementary Video 1.

## Data Availability

The datasets used and/or analyzed during the current study are available from the corresponding author on reasonable request.
